# A Multifractal Vision of 5-Fluorouracil Release from Chitosan-Based Matrix

**DOI:** 10.3390/gels8100661

**Published:** 2022-10-16

**Authors:** Maria-Alexandra Paun, Vladimir-Alexandru Paun, Viorel-Puiu Paun

**Affiliations:** 1 School of Engineering, Swiss Federal Institute of Technology (EPFL), 1015 Lausanne, Switzerland or; 2 Division Radio Monitoring and Equipment, Section Market Access and Conformity, Federal Office of Communications (OFCOM), 2501 Bienne, Switzerland; 3 Five Rescue Research Laboratory, 75004 Paris, France; 4 Physics Department, Faculty of Applied Sciences, University Politehnica of Bucharest, 060042 Bucharest, Romania; 5 Academy of Romanian Scientists, 050085 Bucharest, Romania

**Keywords:** drug release, chitosan, dynamics, non-differentiable scale, multifractal curves

## Abstract

A suite of four drug deliverance formulations grounded on 5-fluorouracil enclosed in a chitosan-founded intercellular substance was produced by 3,7-dimethyl-2,6-octadienal with in situ hydrogelation. The formulations have been examined from a morphological and structural point of view by Fourier transform infrared (FTIR) spectroscopy and microscopy with polarized light, respectively. The polarized optical microscopy (POM) pictures of the three representative formulations obtained were investigated by fractal analysis. The fractal dimension and lacunarity of each of them were thus calculated. In this paper, a novel theoretical method for mathematically describing medicament deliverance dynamics in the context of the polymeric medicament constitution limit has been advanced. Assuming that the polymeric drug motion unfolds only on the so-called non-differentiable curves (considered mathematically multifractal curves), it looks like in a one-dimensional hydrodynamic movement within a multifractal formalism, the drug-release physics models are provided by isochronous kinetics, but at a scale of resolution necessarily non-differentiable.

## 1. Introduction

A topical field of pharmaceutical research is the development of new semi-solid pharmaceutical systems as potential vehicles for the topical release of medicinal substances using excipients of natural origin. The benefits presented by these types of formulations are determined both by the diversity of excipients and by their particularly advantageous specific properties, namely, relative abundance, biocompatibility, biodegradability, non-irritability, innocuousness, and low-cost price. Hydrogels represent an important category of ointment bases, and are frequently used in the formulation of dermatological and cosmetic preparations due to the many advantages they offer. This group also includes biocompatible hydrogels based on natural polymers (e.g., chitosan, pectins, gums) which have been intensively studied in the last decade due to the benefits provided by the specific characteristics of the gel-forming agents.

An initial direction of research was aimed at the development and pharmaco-technical characterization of some hydrogel bases containing a natural hydrophilic polymer, chitosan, as a gelling agent, and different co-solvents able to incorporate and dissolve a large number of medicinal substances insoluble in water. At this stage of the study, the influence of the type and concentration of the co-solvent on the physico-chemical and rheological characteristics of the hydrogel bases with chitosan in different concentrations was evaluated. At the second stage of the experimental research, the possibility of formulating and preparing new biocompatible topical hydrogels based on chitosan together with different natural absorption promoters was investigated.

Chitosan-based pharmaceutical compounds are of great interest in the medicament delivery domain, and are suitable due to their inherent properties of biocompatibility, degradation comportment, and biodegradability, which are advised for current practices in vivo [1,2,3]. In order to further ameliorate chitosan’s capacity to anchor great medicaments’ quantity and to liberate them in a supervised fashion, a lot of tests consisting particularly of chitosan composition crosslinking by different agents have been designed. In this line of conduct, an ecological method was detailed by crosslinking chitosan with eco-friendly products of the monoaldehyde type [4]. The mentioned procedure was found to be a remarkable one, delivering hydrogels whose properties of an aldehyde nature were easily commanded. Therefore, by utilizing the native aldehyde 3,7-dimethyl-2,6-octadienal, well known typically as a right commercial denomination by citral, hydrogels with superior biocompatibility, biodegradability, non-toxic, and non-irritating qualities and with their own antifungal activity were achieved for matrix components for medicament release systems.

Principally, the approach that will be made in the present work, otherwise known as the fractal manner of pharmaco-kinetics (PK) attitude, assumes the utilization of fractal analysis and fractional calculus. Additionally, by increasing the accuracy of performance on the fractal dimension and lacunarity notion, maximum confidence will be achieved when interpreting the images obtained through microscopy. Similarly, it is possible in the context of the analysis of several compartments [5] to successfully describe the polymeric networks’ dynamics and diffusion in porous fractal media. It can be said that, recently, the analysis of several compartments by PK has allowed the modeling of processes such as medication dissolution [6], drug absorption [7], as well as real distribution, together with arranging the constituent parts to achieve the whole [8].

Other things worth mentioning in the introduction are studies that refer to the design of biocompatible chitosan with a high photothermal conversion capability [9], ultrathin fibers and their antibacterial food packaging applications [10] and, last but not least, electrospun structural nanohybrids combining three composites for fast helicopter delivery [11].

In the presented context, special attention was paid to the open problem related to the time development of the kinetics dependent on the major bio-molecular reactions [6]. In continuation, we can say that the compartmental ideal analysis, the newest method for reporting drug delivery dynamics in elaborate systems (operating at a level of fractional derivatives or alternative ordinary procedures utilized in PK), can be depicted, including non-differentiable curves (named multifractal curves). 

Afterwards, as a work alternative with a single mathematical variable represented by a non-differentiable rigid function, it is feasible that we trust only in the approximations of these mathematically defined functions, procured by averaging functions at various scales of determination–resolution. In the aftermath, all proposed variables to depict drug delivery processes will continue to work as the mathematical functions are limited, thus dictating a life with non-differentiable character in the case of scale-zero/nil value resolutions differentiable in different circumstances. Ultimately, the theoretical multifractal model is consequently certified by the empirical experimental data associated with the 5-fluorouracil deliverance out of the chitosan-founded matrix.

## 2. Results and Discussion

### Theoretical Model

Typical investigations on drug-release kinetics deliver significant information on chemical systems’ function. To better explain the subtle transport mechanism and the structure versus function connection of a chemical system, it is decisive to diminish the difference between the experimental macroscopic data and the transport comportment at the atomic and molecular structural level. Obviously, the transport mechanisms at the microscopic level will prevail; these are mechanisms that we also have in mind for the model proposed in the article.

In these circumstances of pharmaco-kinetic behavioral consideration, a novel theoretical method for mathematically describing medicament deliverance kinetics, in the context of the polymeric medicament constitution limit, is advanced [12,13]. Presuming that the polymeric medicament dynamics unfolds only on the so-called non-differentiable curves (considered mathematically multifractal curves), it looks like in a one-dimensional hydrodynamic conventionalism of multifractal arguments, the drug-release physics models are provided by kinetics of synchronous type, but at a scale of resolution necessarily non-differentiable.

The one-dimensional multifractal hydrodynamic equation [11,12,13,14,15] is

(1) $$\frac{\partial }{\partial t}V_{D}+V_{D}\frac{\partial }{\partial x}V_{D}=-\frac{\partial }{\partial x}\left[-2\lambda {\left(dt\right)}^{\left[\frac{4}{f\left(\alpha \right)}\right]-2}\frac{\frac{\partial }{\partial x}(\frac{\partial }{\partial x}\sqrt{\rho )}}{\sqrt{\rho }}\right]$$

(2) $$\frac{\partial }{\partial t}\rho +\frac{\partial }{\partial x}\left(V_{D}\rho \right)=0$$

In the communicated expressions above, *x* is the fractal spatial coordinate (one-dimensional), *t* is the classical (non-fractal) time which plays the role of an affine parameter within the movement curves [14,15]. The speed *V_D_* is the differential velocity (scale resolution dt, independent of time), the function *f*(*α*) is the singularity spectrum, *α* is dependent, and √*ρ* is a function of the state’s amplitude.

The initial conditions (3) and boundary conditions (4) of the above equations are

(3) $$V_{D}\left(x,t=0\right)=V_{0},\;\;\rho \left(x,t=0\right)=\frac{1}{\sqrt{\pi }\alpha }exp\left[-{\left(\frac{x}{\alpha }\right)}^{2}\right]$$

(4) $$V_{D}\left(x=V_{0}t\right)=V_{0},\;\;\rho \left(x=-\infty ,t\right)=\rho \left(x=+\infty ,t\right)=0$$

where $V_{0}$ is the initial speed, while α is the Gaussian distribution parameter of the position variable x. 

According to the mathematical procedures recommended in bibliographical references [16], differential Equations (1) and (2) accept the following solutions

(5) $$V_{D}\left(x,t,\sigma ,\alpha \right)=\frac{V_{0}\alpha^{2}+{\left(\frac{\sigma }{\alpha }\right)}^{2}xt}{\alpha^{2}+{\left(\frac{\sigma }{\alpha }\right)}^{2}t^{2}}$$

(6) $$\rho \left(x,t,\sigma ,\alpha \right)=\frac{\pi^{-1/2}}{{\left(\alpha^{2}+{\left(\frac{\sigma }{\alpha }\right)}^{2}t^{2}\right)}^{1/2}}\exp\left[-\frac{{\left(x-V_{0}t\right)}^{2}}{\alpha^{2}+{\left(\frac{\sigma }{\alpha }\right)}^{2}t^{2}}\right]$$

where

(7) $$\sigma =\lambda {\left(dt\right)}^{\left[\frac{2}{f\left(\alpha \right)}\right]-1}$$

is the multifractal degree. 

According to the stated fractal theory, the non-differentiable velocity $V_{F}$ will have the formula

(8) $$V_{F}\left(x,t,\sigma ,\alpha \right)=\sigma \frac{\left(x-V_{0}t\right)}{\alpha^{2}+{\left(\frac{\sigma }{\alpha }\right)}^{2}t^{2}}$$

By inserting the dimensionless variables in the above expressions

(9) $$\xi =\frac{x}{V_{0}\tau_{0}},\;\;\;\eta =\frac{t}{\tau_{0}}$$

as well as the dimensionless parameters

(10) $$\mu =\frac{\sigma \tau_{0}}{\alpha^{2}},\;\;\;\phi =\frac{\alpha }{V_{0}\tau_{0}}$$

where $\tau_{0}$ is named specific time. Formulas (5), (6) and (8) are written as

(11) $$V\equiv V_{D}\left(\xi ,\eta ,\mu \right)=\frac{V_{D}\left(x,t,\sigma ,\alpha \right)}{V_{0}}=\frac{1+\mu^{2}\xi \eta }{1+\mu^{2}\eta^{2}}$$

(12) $$\rho \left(\xi ,\eta ,\mu ,\phi \right)=\pi^{\frac{1}{2}}\alpha \rho \left(x,t,\sigma ,\alpha \right)={\left(1+\mu^{2}\eta^{2}\right)}^{-\frac{1}{2}}exp\left[-\frac{{\left(\xi -\eta \right)}^{2}}{\phi^{2}\left(1+\mu^{2}\eta^{2}\right)}\right]$$

(13) $$U\equiv V_{F}\left(\xi ,\eta ,\mu \right)=\frac{V_{F}\left(x,t,\sigma ,\alpha \right)}{V_{0}}=\mu \frac{\left(\xi -\eta \right)}{1+\mu^{2}\eta^{2}}$$

**Figure 1:**
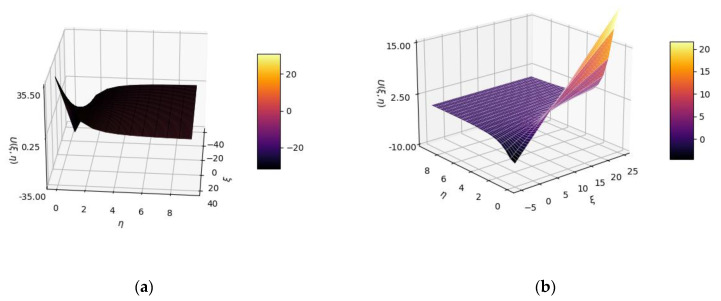
Three-dimensional plot representations of U(ξ,η) multifractal function for two distinct sets of variable values *ξ* and *η*: (**a**) *ξ* from −40 to 40; *η* from 0 to 10; (**b**) *ξ* from −5 to 25; *η* from 0 to 10.

In Figure 1a,b, 3D plot representations of the U(ξ,η) multifractal function are presented for two distinct sets of variable values *ξ* and *η*, utilized for drug-release analysis.

**Figure 2:**
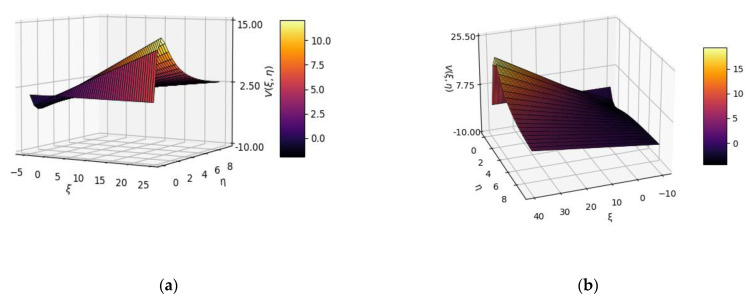
Three-dimensional plot representations of V(ξ,η) multifractal function for two distinct sets of variable values *ξ* and *η*: (**a**) *ξ* from −5 to 25; *η* from 0 to 10; (**b**) *ξ* from −40 to 40; *η* from 0 to 10.

In Figure 2, 3D plot representations of the V(ξ,η) multifractal function are presented for two distinct sets of variable values *ξ* and *η*, utilized for drug-release analysis.

If we now make the ratio between *U* and *V*, respectively, *U*/*V*, we obtain a homographic expression dependent of *ξ* and *η*, as presented below:

(14) $$\frac{U}{V}=\frac{\mu \left(\xi -\eta \right)}{1+\mu^{2}\xi \eta }$$

**Figure 3:**
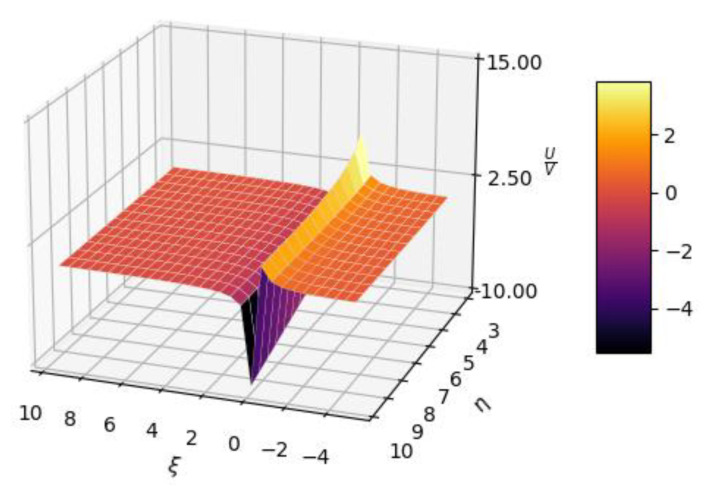
Three-dimensional plot representations of *U*/*V* multifractal function.

In Figure 3, 3D plot representations of the *U*/*V* multifractal function, with expressions dependent of the *ξ* and *η* variables, are presented.

In the case of the condition validity in which the differential of the ratio *U*/*V* is zero (so-called dynamic simultaneity)

(15) $$d\left(\frac{U}{V}\right)=\frac{dU-dV}{V^{2}}=0\Leftrightarrow V=constU$$

we have between *U* and *V* the relationship *V* = *constU*.

This coincides with the prolongation of the Newton first principle to any scale resolution, or similarly, with to the “synchronizations” of drug-release dynamics at a non-differentiable scale. Additionally, it can be mentioned that the varied “mechanisms” implicated in drug-release activity may be mimicked by doubling the period, or a manifested quasi-periodicity and the process’ intermittent solicitations [15].

Following the activation of Restriction (15), in the formulation *V*=-*U* and Equations (1) and (2), the multifractal-type conservation laws become a unique multifractal equation, a law of “diffusion” type

(16) $$\partial_{t}\rho =\lambda {\left(dt\right)}^{\left[\frac{2}{f\left(\alpha \right)}\right]-1}\partial_{l}\partial^{l}\rho =\sigma \partial_{l}\partial^{l}\rho$$

As an immediate result, these evidentiated mechanisms manifests themselves as diffusion processes in a multifractal space but at diverse scale resolutions, which can be at large classified in the category of Fickian diffusion (when polymer relaxation time *t_r_* is much greater than the value *t_d_*, characteristic solvent diffusion time) or, as non-Fickian-type diffusion (when *t_r_* ≈ *t_d_*), with the same ease. If the medicament release is of the multifractal type and takes place in a perfect immersed state, the initial conditions and conditions at the boundary are accepted

(17) $$t=0,\;-\frac{\alpha }{2}<x<\frac{\alpha }{2},\;\;\rho =\rho_{0}\;t>0,\;\;x=\pm \frac{\alpha }{2},\;\;\rho =\rho_{1}$$

wherein $\rho_{0}$ is the primary drug density of the multifractal-type states, as part of the fractal-type “mechanism” used, and $\rho_{1}\;$is the current medicament density according to polymer–fluid fractal interdependence. The equation solution in the given conditions may now be presented in the complex form as below [16]:

(18) $$f=\frac{\rho_{t}}{\rho_{\infty }}=2{\left(\frac{\sigma t}{\delta^{2}}\right)}^{\frac{1}{2}}=\left\{\pi^{-1/2}+\overset{\infty }{\underset{n=1}{\sum }}{\left(-1\right)}^{n}erfc\left[\frac{n\delta }{2{\left(\sigma t\right)}^{\frac{1}{2}}}\right]\right\}$$

A precise expression was achieved for little values of time *t*

(19) $$f=\frac{\rho_{t}}{\rho_{\infty }}=2{\left(\frac{\sigma t}{\delta^{2}}\right)}^{\frac{1}{2}}=const{\left(t\right)}^{\frac{1}{2}}$$

In these circumstances, the ratio $\frac{\rho_{t}}{\rho_{\infty }}$ can be approved as an equivalent dissolved drug fraction, i.e., $\frac{M_{t}}{M_{\infty }}\equiv \frac{\rho_{t}}{\rho_{\infty }}$, where *M_t_* is the drug quantity dissolved in time *t* and $M_{\infty }$ is the drug quantity dissolved in the total time, considering that the pharmaceutical graduation shape has been already exhausted.

**Figure 4:**
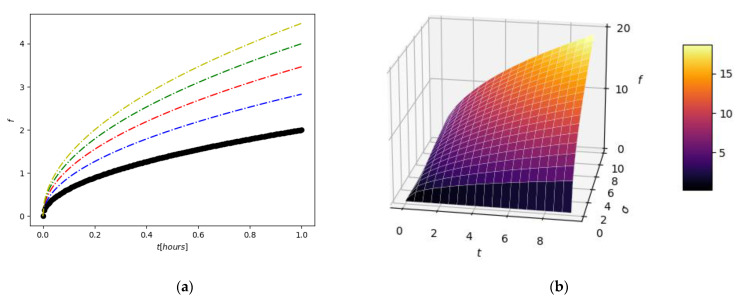
Two-dimensional contour (**a**) and three-dimensional plot (**b**) representations of *f* multifractal function.

The function *f* depends on *t*, and *σ* plays the role of the parameter. In Figure 4, a 2D curve is obtained for a fixed value of the parameter. Thus, the black color represents the value *σ* = 1, the blue color represents *σ* = 2, the red color represents *σ* = 3, the green color represents *σ* = 4, and the olive color represents *σ* = 5. In Figure 4, a 3D function *f* = *f* (*t*, *σ*) is considered by three variables; one allows the variation of *σ* continuously with a parameter value between 0 and 10.

**Figure 5:**
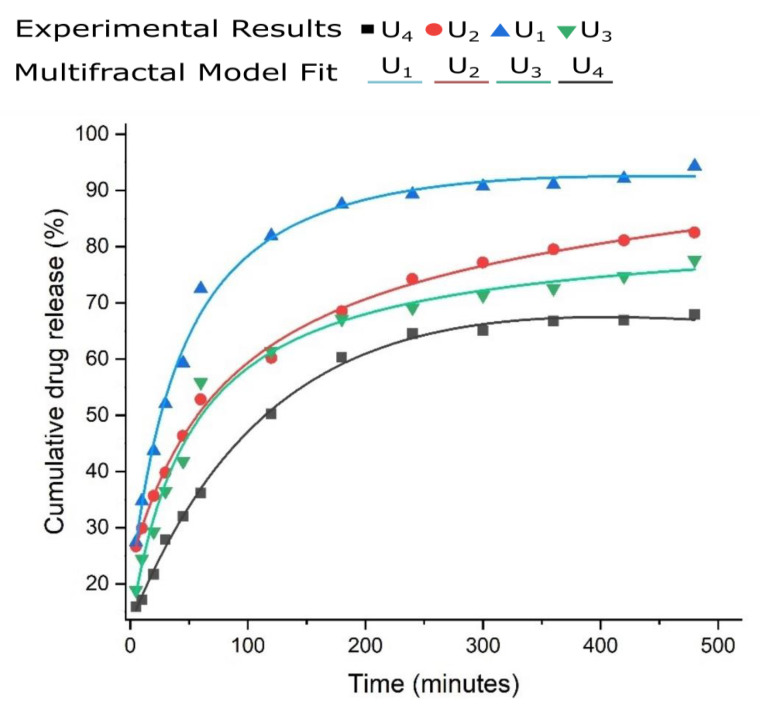
5-fluorouracil release of experimental presentation [17]; the formulations are accommodated in multifractal theoretical replica (solid-colored lines).

In Figure 5, a model confirmation of the 5-fluorouracil release out of the chitosan-founded matrix is depicted. The experimental results are fit tightly with the mathematical theoretical functions of multifractal conception replica. The obtained graphical figure demonstrated that the realized model is ready to successfully prophesy the drug-deliverance kinetics [15,16].

The legend of Figure 5, with the symbols for defining the experimental points and the colors for each type of fractal curve used in the case of the four tested substances, is presented above. As can be seen in Figure 5, from the graphic representation of cumulative drug release as a function of time, curves U_1_ and U_4_ are classic saturation curves, while curves U_2_ and U_3_ are increasing with time (tend to infinite values). 

## 3. Evaluation of Polarized Optical Microscopy Pictures by Fractal Analysis

**Figure 6:**
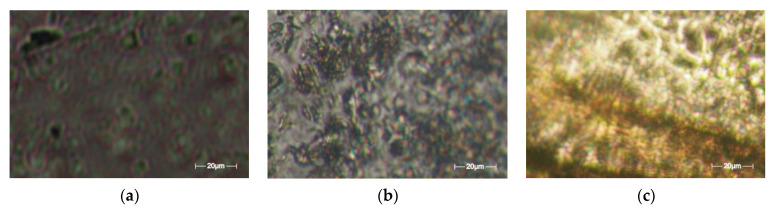
Representative POM images [17] of the formulations (**a**) U_1_; (**b**) U_2_; (**c**) U_4_.

5-fluorouracil’s existence in chemical formulations was confirmed by means of polarized light microscopy (Figure 6). In the provided images, the obvious drug segregation into the hydrogels is clearly visible, with great density of crosslinking in compounds U_1_ and U_2_, in contrast with the hydrogel formulations of reduced crosslinking density (U_4_), about which several things are discussed. Thus, a birefringent comportment and a granular texture were declared; a specific feature of the crystal is its sub-micrometric dimensions, but it still falls below the detection limit of the analysis devices used [17].

**Figure 7:**
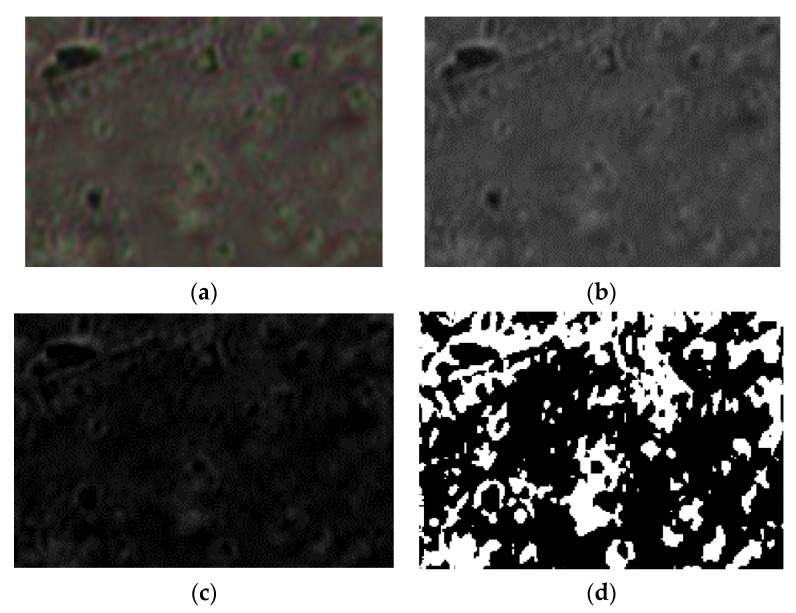
Primary processing of the selected image U_1_. (**a**)—original image (the entire portion); (**b**)—the grayscale version; (**c**)—the gray scale version without luminance; (**d**)—binarized version. A threshold of 14 was used for binarization.

The scale bar for the POM photographic images (from Figure 6: (a) U_1_; (b) U_2_; (c) U_3_) is 20 microns. The POM image in Figure 7 of experimentally produced formulations were processed according to fractal analysis standards, calculating the fractal dimension and lacunarity of each one. The processing method and the values obtained for fractal dimension and lacunarity, together with the voxel representation for each image separately are presented below, in paper continuation.

The fractal dimension and the lacunarity of the investigated POM images were calculated according to known fractal analysis procedures [18,19]. The same advanced mathematical utilities were successfully used in a previous work, but for images obtained by scanning electron microscopy (SEM), which will prove to be a complementary reading [20]. In order to obtain a high resolution, the calculation programs/software developed for the evaluation of some medical diseases with CT and MRI images were used. It is known that significant differences are noticed at the pixel level in order to give an early quantitative diagnosis (see [21,22,23]).

Following the numerical evaluations with the appropriate software of the selected image U_1_, the values of fractal dimension *D* = 1.7602, standard deviation $s=\pm \sqrt{\sigma^{2}}=\pm 0.2026$ (see [24]) and lacunarity Λ=0.0215 were obtained [24,25,26], as in Table 1.

**Figure 8:**
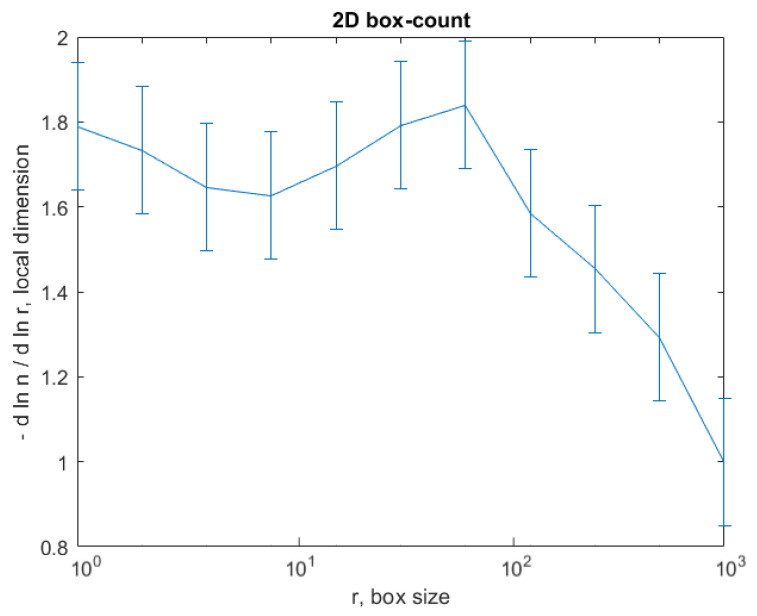
Box-count algorithm results: fractal dimension.

The graph in Figure 8 shows the results of the 2D box-count algorithm and the local fractal dimension of the investigated image U_1_ [25,27].

**Figure 9:**
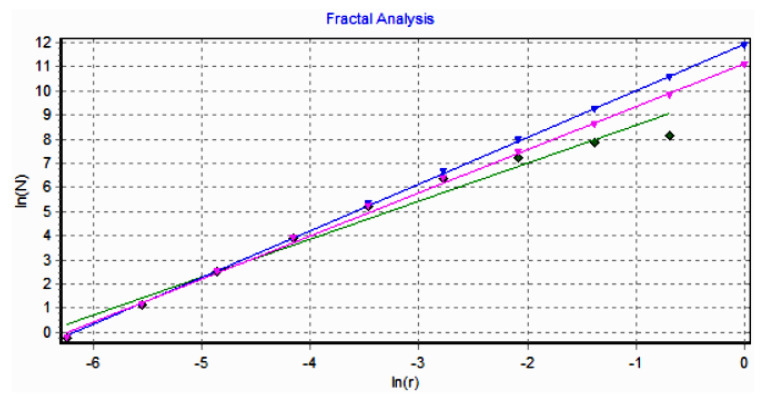
Graphic of fractal dimension for selected U_1_ image area.

Figure 9 shows the verification of the selected U_1_ image area with the software Harmonic and Fractal Image Analyser Demo version 5.5.30 [28] of the fractal dimension for various ruler scales *r*.

**Figure 10:**
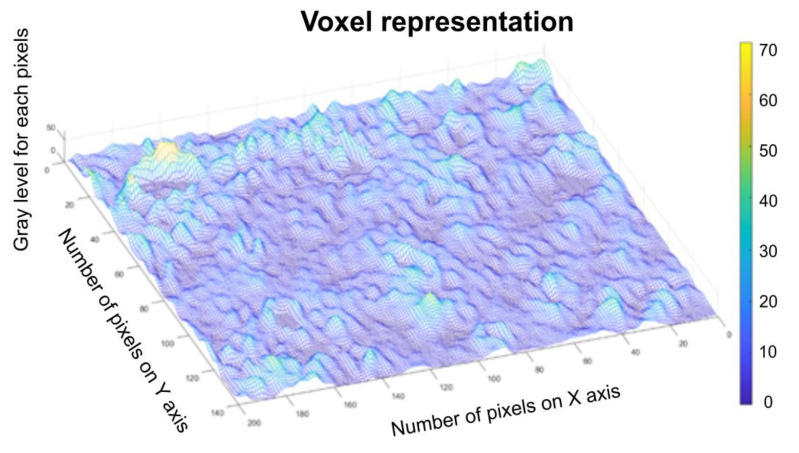
Voxel representation for the image U_1_.

Figure 10 represents a three-dimensional graph of the voxel representation for the image U_1_ from the modified area.

**Figure 11:**
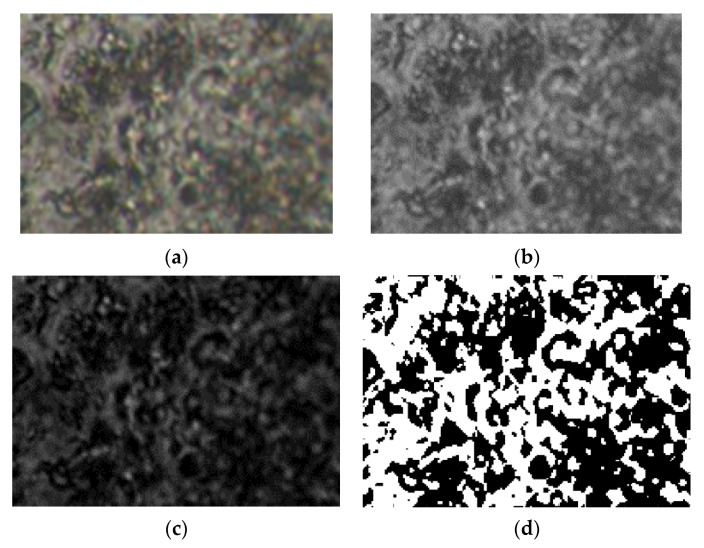
Primary processing of the selected image U_2_. (**a**)—original image (the entire portion); (**b**)—the grayscale version; (**c**)—the gray scale version without luminance; (**d**)—binarized version. A threshold of 25 was used for binarization.

The POM image in Figure 11 of experimentally produced formulations were processed according to fractal analysis standards, calculating the fractal dimension and lacunarity of each one.

Following the numerical evaluations with the appropriate software of the selected image U_2_, the values of fractal dimension *D* = 1.7523, standard deviation $s=\pm \sqrt{\sigma^{2}}=\pm 0.1949$ (see [24]) and lacunarity Λ=0.0363 were obtained [24,25,26], as in Table 2.

**Figure 12:**
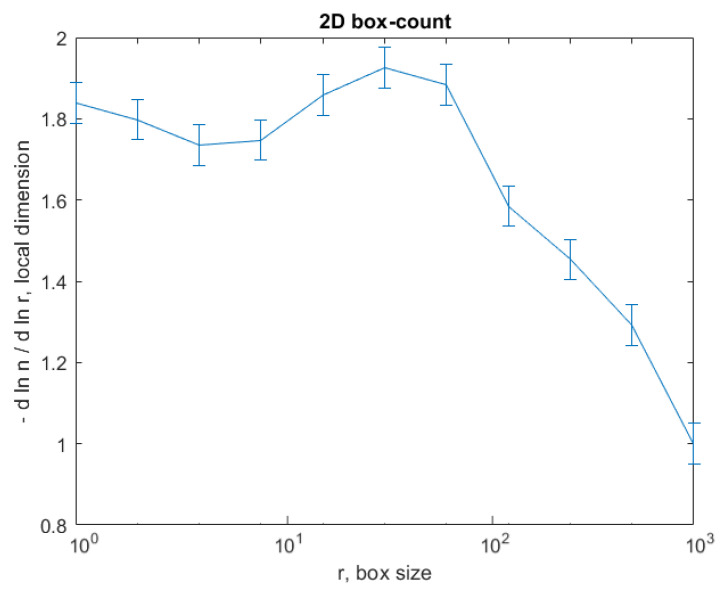
Box-count algorithm results: fractal dimension.

The graph in Figure 12 shows the results of the 2D box-count algorithm and the local fractal dimension of the investigated image U_2_ [25,27].

**Figure 13:**
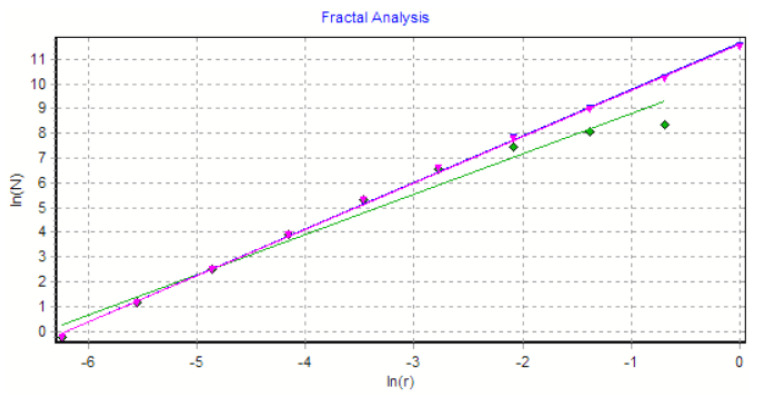
Graphic of fractal dimension for selected U_2_ image area.

Figure 13 shows the verification of the selected U_2_ image area with the software Harmonic and Fractal Image Analyser Demo version 5.5.30 [28] of the fractal dimension for various ruler scales *r*.

**Figure 14:**
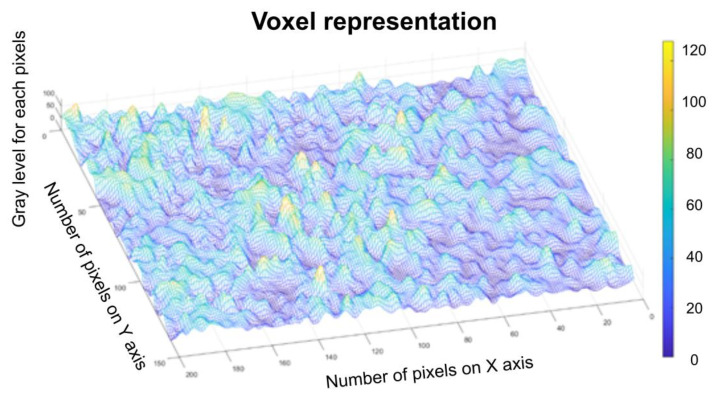
Voxel representation for the image U_2_.

Figure 14 represents the three-dimensional graph of voxel representation for the image U_2_ from the modified area.

Following the numerical evaluations with the appropriate software of the selected image U_4_, the values of fractal dimension *D*=1.7352, standard deviation $s=\pm \sqrt{\sigma^{2}}=\pm 0.1831$ (see [24]) and lacunarity Λ=0.0385 were obtained [24,25,26], as in Table 3. 

**Figure 15:**
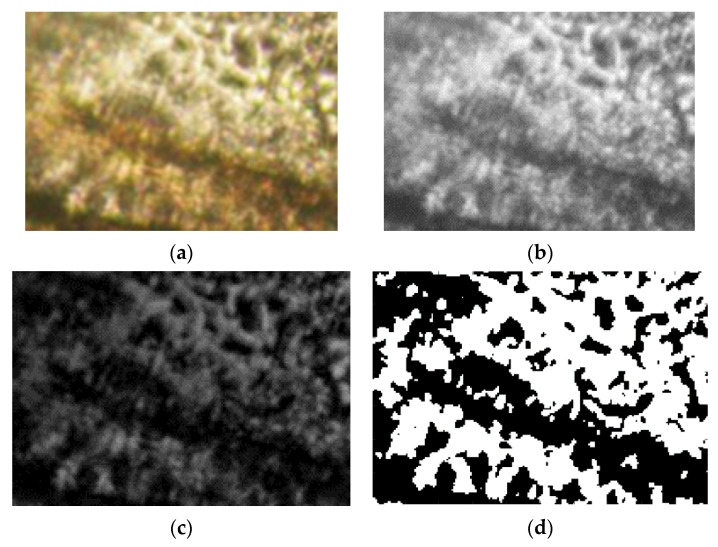
Primary processing of the selected image U_4_. (**a**)—original image (the entire portion); (**b**)—the grayscale version; (**c**)—the gray scale version without luminance; (**d**)—binarized version. A threshold of 35 was used for binarization.

Figure 7a–d, Figure 11a–d and Figure 15a–d show the three transformations (b, c and d) of the original of Figure 7a, Figure 11a and Figure 15a. Thus, the following changes were obtained: (b) the grayscale version; (c) the gray scale version without luminance; (d) the binarized version. The thresholds of 14, 25, and 35 were used for binarization. In [Table 1, Table 2 and Table 3], the values of fractal dimension, standard deviation and lacunarity that were obtained following the evaluation of images U_1_, U_2_ and U_3_ from the original Figure 7a, Figure 11a and Figure 15a are shown.

The POM image in Figure 15 of experimentally produced formulations were processed according to fractal analysis standards, calculating the fractal dimension and lacunarity of each one.

**Figure 16:**
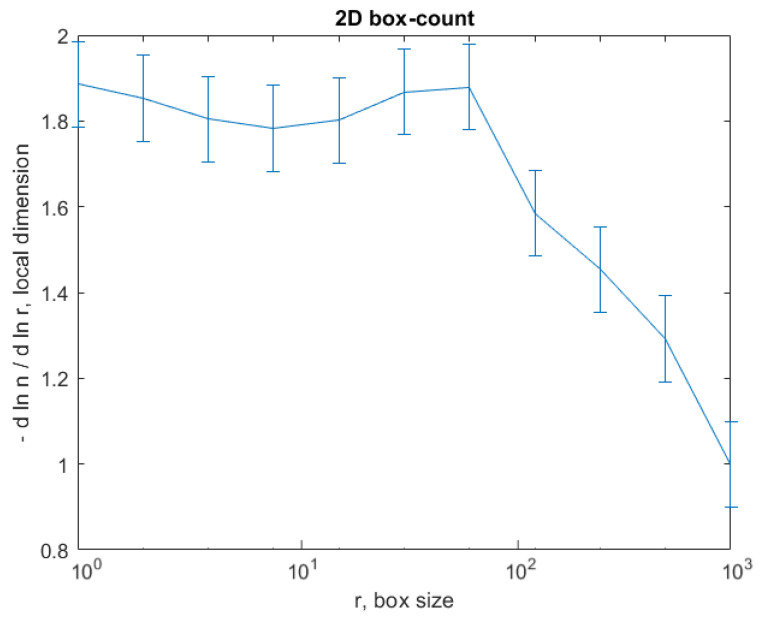
Box-count algorithm results: fractal dimension.

The graph in Figure 16 shows the results of the 2D box-count algorithm and the local fractal dimension of the investigated image U_4,_ [25,27].

**Figure 17:**
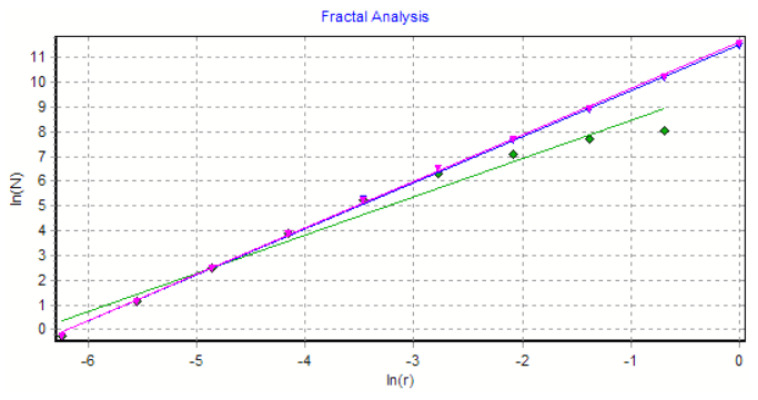
Graphic of fractal dimension for selected U_4_ image area.

Figure 17 shows the verification of the selected U_4_ image area with the software Harmonic and Fractal Image Analyser Demo version 5.5.30 [28] for the fractal dimension with various ruler scales *r*.

**Figure 18:**
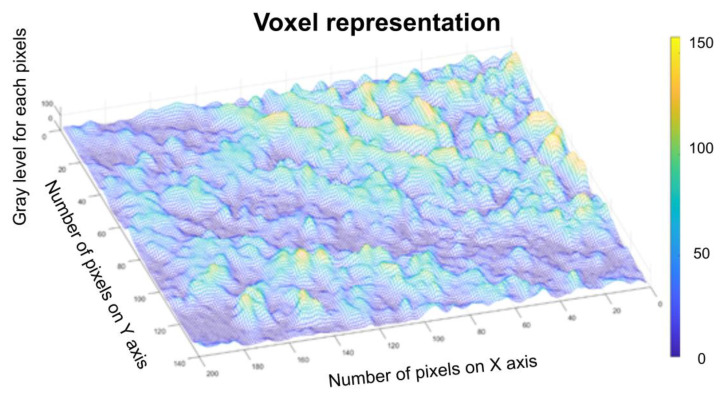
Voxel representation for the image U_4_.

Figure 18 represents the three-dimensional graph of the voxel representation for the image U_4_ from the modified area. 

Figure 8, Figure 9, Figure 12, Figure 13, Figure 16 and Figure 17 present the graphic representations of the 2D fractal dimension used for the linear regression slope calculation of the fractal dimension. Figure 10, Figure 14 and Figure 18 represent the three-dimensional graphs of voxel representation for the images U_1_, U_2_ and U_4_ from the modified area. The three coordinate axes are distributed as follows: the number of pixels on the ox axis, the number of pixels on the oy axis, and the gray level for each pixel on the oz axis. In 3D computer graphics, the voxel represents a numerical value associated with the regular grid of a three-dimensional coordinate space.

## 4. Conclusions

Suites of drug delivery arrangements were realized by 5-fluorouracil encapsulation in the hydrogel matrix configured by the reticulation of chitosan-based with 3,7-dimethyl-2,6-octadienal. The hydrogel demonstrated a special capability to anchor the medicament, ensuring its prolonged deliverance close to 24 h. The release speed was adjusted by fluctuating the crosslinking consistency, arriving at 90% for a great reticulation density and at 70 % for a reduced one. In the stated multi fractal paradigm, a mathematical model was evolved for the comprehension of the drug delivery dynamics, taking into account that these special comportments are closely related to the fact that the movement takes place on non-differentiable curves. 

Worth mentioning is the fact that, for the one-dimensional case of the multifractal hydrodynamic formalism, the ratio between the differentiable and non-differentiable velocities of a distinct distance is contingent on time, in a homographic fashion. The simultaneous dynamic conditions suggest the synchronization of the drug deliverance techniques at both scale resolutions, formulated by the multifractal diffusion functions, since we have the diffusion process depending on the scale resolutions. The fitted curves of the theoretical model are in good agreement with the obtained experimental results.

The three images of the U_1_, U_2_ and U_4_ compounds, obtained by means of polarized light microscopy, were investigated according to the standards of fractal analysis. Thus, for image U_1_, we found the fractal dimension D = 1.7602 ± 0.2026 and lacunarity Λ=0.0215; for image U_2_, we found the fractal dimension D = 1.7523±0.1949 and lacunarity Λ=0.0363; and for Image U_4_, we found the fractal dimension D = 1.7352±0.1831 and lacunarity Λ=0.0385. 

## 5. Materials and Methods

### 5.1. Materials

From the point of view of chemistry, the chitosan (CS) is a basic polysaccharide acquired by the deacetylation of chitin, one of the most plentiful native biopolymers in the world, with the exception of cellulose, evidently. In large part due to its high biocompatibility, biodegradability and reduced toxicity, chitosan has been utilized on a large scale in the pharmaceutical industry. In the last decade, chitosan utilization as a conveyor of continuous-release preparations to resolve the half-life period of chemotherapy medicaments has been announced [29]. In addition, chitosan-covered magnetic nanoparticles were utilized to extend the liberation of 5-fluorouracil (5-FU) [4]. CS (243 kDa, DA: 87%), 3,7-dimethyl-2,6-octadienal (95%), 5-FU, and phosphate tampon solution with a pH of 7.4 were acquisitioned from Aldrich chemical company and utilized as they were received.

### 5.2. Synthesis of the Hydrogel Formulation Preparations

The formulations used were fabricated through the hydrogelation in situ method of chitosan (CS) with 3,7-dimethyl-2,6-octadienal in the company of 5-fluorouracil (5-FU), which is a respected and classic protocol [3,4]. In a very short time, a 2% solution of 3,7-dimethyl-2,6-octadienal combined with 5-fluorouracil was gently dripped into a 3% chitosan-dissolved solution in aqueous acetic acid solution (1%). All substances were fabricated by Aldrich chemical company. 

According to the observations made, it was accepted that 5-fluorouracil was fixed in the matricial structure as a crystalline component, and its size was assorted with the crosslinking density value. The in vitro drug release simulated in an environment that imitates the physiological habitat disclosed a 5-fluorouracil gradual deliverance, in close relation with the value of crosslinking density [23]. 

The drug and chitosan quantity were both retained steadily, while the 3,7-dimethyl-2,6-octadienal amount was diversified to touch various molar proportions of the amine/aldehyde groups, from the ratio of 1/1 to 4/1, in order to achieve hydrogels with different values of crosslinking consistency [7]. In the end, it was found that the hydrogelation period increased as the aldehyde quantity decreased. Thereby, the 1/1 molar ratio of amine/aldehyde functional classes immediately took place and continued sluggishly for 24 h for the 4/1 molar ratio of the same classes. Eventually, the acquired hydrogels were lyophilized and subjected to detailed analysis. Moreover, for the inventory, the formulations received the labels U_1_, U_2_, U_3_, U_4_, where the attached number was in agreement with the amino/aldehyde classes’ molar ratio. 

### 5.3. Methods

The gelation period was visually established in the moment when the reaction blend was transformed out of its initial viscous state to a rubbery final state. We mention that the xerogels were realized by lyophilization of the adequate hydrogels, utilizing the Labconco FreeZone Freeze Dry System, (FreeZoner2.5 Liter Freeze Dry Systems) instrument, for a duration of 24 h at a temperature of −50 °C and pressure of 0.04 mbar. The formulations produced were described by FTIR spectroscopy using the FT-IR Bruker Vertex 70 Spectrophotometer(Art photonics GmbH, 12489 Berlin, Germany).

The drug-release kinetics of the detailed medicaments’ delivery systems were identified by recording the absorbance at 265 nm from the supernatant where the release was completed. The concentration of the solution was computed with the help of the Beer–Lambert law. The supernatant UV–vis spectra were recorded on a Horiba spectrophotometer, but the absorbance was accommodated on a previously drawn calibration curve. The 5-fluorouracil curve of calibration was drawn utilizing the absorption maximum spectrum at 265 nm.

## Figures and Tables

**Table 1:** Calculation of fractal parameters for picture U_1_.

Name	Fractal Dimension	Standard Deviation	Lacunarity
Image U_1_	1.7602	±0.2026	0.0215

**Table 2:** Calculation of fractal parameters for picture U_2_.

Name	Fractal Dimension	Standard Deviation	Lacunarity
Image U_2_	1.7523	±0.1949	0.0363

**Table 3:** Calculation of fractal parameters for picture U_4_.

Name	Fractal Dimension	Standard Deviation	Lacunarity
Image U_4_	1.7352	±0.1831	0.0385

## Data Availability

The data used to support the findings of this study cannot be accessed due to commercial confidentiality.
